# Polypharmacy, Potentially Inappropriate Medications, and Drug-to-Drug Interactions in Patients with Chronic Myeloproliferative Neoplasms

**DOI:** 10.3390/biomedicines11051301

**Published:** 2023-04-27

**Authors:** Ivan Krečak, Ljerka Pivac, Marko Lucijanić, Marko Skelin

**Affiliations:** 1 Department of Internal Medicine, General Hospital of Sibenik-Knin County, 22000 Sibenik, Croatia; 2 Faculty of Medicine, University of Rijeka, 51000 Rijeka, Croatia; 3 Pharmacy Department, University Hospital Center Split, 21000 Split, Croatia; 4 Divison of Hematology, University Hospital Dubrava, 10000 Zagreb, Croatia; 5 Faculty of Medicine, University of Zagreb, 10000 Zagreb, Croatia; 6 Pharmacy Department, General Hospital of Sibenik-Knin County, 22000 Sibenik, Croatia

**Keywords:** myeloproliferative neoplasms, polypharmacy, drug interactions, survival, thrombosis, polypharmacy, potentially inappropriate medications, drug-to-drug interactions

## Abstract

Polypharmacy, potentially inappropriate medications (PIMs), and drug-to-drug interactions (DDIs) are highly prevalent in the elderly and may have adverse effects on health-related outcomes. Their occurrence and clinical and prognostic associations in patients with chronic myeloproliferative neoplasms (MPN) are unknown. We retrospectively evaluated polypharmacy, PIMs, and DDIs in a cohort of 124 MPN patients (essential thrombocythemia, ET = 63, polycythemia vera, PV = 44, myelofibrosis = 9, MPN unclassifiable = 8) from a single community hematology practice. There were 761 drug prescriptions with a median of five prescribed medications per patient. Polypharmacy, at least one PIM (calculated for persons >60 years of age, *n* = 101), and at least one DDI were recorded in 76 (61.3%), 46 (45.5%), and 77 (62.1%) of patients, respectively. Seventy-four (59.6%) and twenty-one (16.9%) patients had at least one C or at least one D interaction, respectively. Among other associations, polypharmacy and DDIs were associated with older age, management of disease-related symptoms, osteoarthritis/osteoporosis, and different CV disorders. In multivariate analyses adjusted for clinically meaningful parameters, both polypharmacy and DDIs were significantly associated with inferior overall survival (OS) and time to thrombosis (TTT), whereas PIMs had no significant associations with neither OS nor TTT. There were no associations with bleeding or transformation risks. Polypharmacy, DDIs, and PIMs are very frequent among MPN patients and may have important clinical associations.

## 1. Introduction

Philadelphia chromosome-negative myeloproliferative neoplasms (MPNs), essential thrombocythemia (ET), polycythemia vera (PV), and myelofibrosis (MF), share several clinical and biological characteristics; these include the overproduction of erythroid, megakaryocytic and granulocytic cells, frequent splenomegaly, variable degrees of bone marrow fibrosis, and an increased thrombohemorrhagic risk [1,2]. The majority of MPN patients bear mutually exclusive driver mutations in the *Janus kinase 2* (*JAK2*) [3,4] or *calreticulin* (*CALR*) genes [5,6] that constitutively activate the JAK-STAT signaling pathway which causes excessive myeloproliferation and a persistent chronic inflammatory state response responsible for the frequent constitutional symptoms associated with the disease [7,8]. Life expectancy in ET and PV is worse than in the general population, mainly due to adverse cardiovascular (CV) events and disease transformation to secondary MF (SMF), myelodysplastic syndrome (MDS), or acute myeloid leukemia (AML) [9,10]. The median survival in MF patients is the worst among the three MPNs and is approximately 6 to 7 years; death usually occurs because of disease progression and bone marrow failure [11]. Interestingly, thrombotic risk may persist even after the transformation of ET and PV into SMF [12]. Therefore, the main therapeutic goals in MPNs are to mitigate thrombohemorrhagic risk and to postpone disease progression to prolong patient survival. Age >60 years and/or prior thrombosis have been recognized as the most important risk factors for both thrombosis and survival in ET and PV [1,13], whereas advanced age, leukocytosis, anemia, presence of peripheral blasts, and constitutional symptoms are the main predictors of inferior survival in MF patients [2]. In ET, the presence of the *JAK2* mutation was shown to be more thrombogenic than the presence of the *CALR* mutation [1]. Even though stringent control of CV risk factors is recommended by the current therapeutic guidelines [13], the exact magnitude of the effect of different CV risk factors, such as arterial hypertension [14], hyperlipidemia [15], hyperuricemia [16,17], chronic kidney disease [18,19,20], smoking [21], or leukocytosis [22] on thrombotic risk and survival in MPNs remains uncertain [23,24]. On the other hand, bleeding in MPNs is less frequent than thrombosis and may occur in up to 20% of patients. Its risk factors are less well-defined; the most important ones seem to be advanced age, prior bleeding, MF phenotype, splenomegaly, thrombocytopenia, acquired von Willebrand disease, and antiplatelet/anticoagulant use [25,26,27]. 

Interferons and hydroxyurea are recommended as first-line treatments for high-risk ET and PV patients, whereas ruxolitinib, a *JAK1/2* inhibitor, is used for the initial treatment of intermediate/high-risk MF patients [1,2,13]. All PV patients receive low-dose aspirin and are additionally periodically phlebotomized to maintain the hematocrit <45%, as this intervention has been shown to lower the rates of adverse CV events. Low-dose aspirin is also recommended for low- or intermediate-risk ET patients who are *JAK2*-positive or with other CV risk factors [1,13].

Polypharmacy describes the use of multiple medications to treat different medical conditions in an individual; its prevalence continues to grow over time and with the patient’s age [28,29]. The use of multiple medications is also strongly associated with CV pharmacotherapy in the elderly due to frequent CV multimorbidity [30]. On the other hand, polypharmacy may increase the risk of adverse drug reactions and drug-to-drug interactions (DDIs) which may cause increased morbidity, mortality, and healthcare costs [31]. Similarly, potentially inappropriate medications (PIMs), defined as ineffective medicines and/or medicines with a high risk-to-benefit ratio, have become a global public health concern due to their high prevalence in the elderly and their adverse effect on health-related outcomes. [32,33]. Considering that MPNs are usually diagnosed in the elderly and are burdened with disease-related symptoms and a high CV risk, this study aimed to investigate the prevalences of polypharmacy, PIMs, and DDIs in MPN patients, their clinical associations, and the impact on survival and thrombohemorrhagic and disease transformation risk. 

## 2. Patients and Methods

### 2.1. Study Design and Patient Population

**Figure 1:**
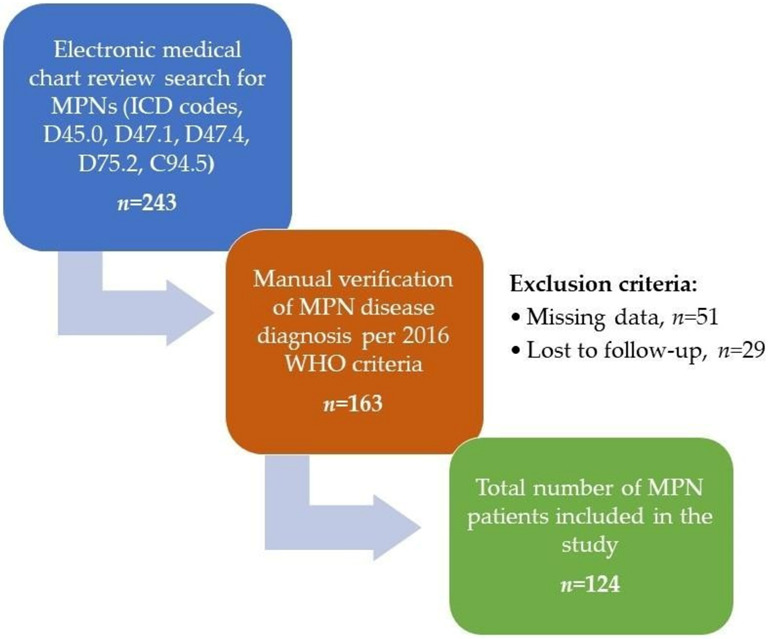
Study flowchart. MPNs = myeloproliferative neoplasms, ICD = International Classification of Diseases, WHO = World Health Organization.

This was a single-center study conducted at the General Hospital of Sibenik-Knin County, Sibenik, Croatia in the period between January 1996 and November 2022. Patients with MPNs were retrospectively identified through medical chart review and the details regarding demographic, clinical, and laboratory data were collected at the time of disease diagnosis or at the time of first patient referral. ET, PV, and MF disease diagnoses were first extracted from the medical records using the International Classification of Diseases (ICD), 10th revision, codes for MPNs (D45.0, D47.1, D47.4, D75.2, C94.5) which were then manually verified and reassessed according to World Health Organization (WHO) 2016 criteria [34]. For patients diagnosed before 2005 (when the *JAK2-V617F* mutation was first discovered), this mutation analysis was performed patients when it became available. *CALR* mutations were performed in a smaller proportion of ET and MF patients after it became available in Croatia (2016). Patients lost to follow-up or with missing data were excluded. The study flowchart is presented in Figure 1. 

Disease-related symptoms were defined as fatigue, night sweats, weight loss (>10% of body weight in the preceding 6 months), fevers, pruritus, lack of concentration, headaches, and early satiety. Cardiovascular risk factors of interest were arterial hypertension (defined as arterial blood pressure >140/90 mmHg or the use of antihypertensives), hyperlipidemia (total cholesterol >5 mmol/L and/or low-density lipoprotein levels >3 mmol/L or the use of antilipemics), diabetes mellitus (diagnosed by an endocrinologist), smoking (active/prior vs. never smoker) and chronic kidney disease (estimated glomerular filtration rate < 60 mL/min/1.73 m^2^ ≥ 3 months). Chronic heart failure (CHF) was defined as left ventricular ejection fraction < 50% or the need for diuretics to keep euvolemia. Hyperuricemia was defined as serum uric acid >428.26 µmol/L for adult males and >356.88 µmol/L for adult females [35].

A total number of prescriptions was determined for the entire follow-up. We used Anatomical Therapeutic Chemical Classification (ATC) system developed by the WHO to stratify medications into 14 categories according to their therapeutical and chemical characteristics (URL: http://www.whocc.no/atc/structure_and_principles/; accessed on 10 February 2023). Polypharmacy, PIM, and DDI were defined at study entry, and patients stratified as such did not change their status during the study follow-up. Polypharmacy marked the concomitant use of ≥5 medicines and PIMs were classified according to EU(7)-PIM list; the latter tool is recommended to screen for medications in elderly persons (>60 years of age) which should be avoided due to a high risk of adverse events and/or insufficient evidence of their benefit and when there are equally or more effective but lower risk alternatives available [36]. DDIs were stratified using Lexicomp^®^ (Lexi-Drug Interaction Online; UpToDate, Inc.: Hudson, OH, USA) [37]. This online software classifies DDI into five categories; A (no interaction), B (no action needed), C (monitor therapy), D (modify regimen), and X (avoid combination). Considering the generally harmless nature of A and B interactions and for the purpose of this analysis, we focused solely on C, D, and X interactions. 

### 2.2. Statistics

According to Shapiro–Wilk’s test, the data were not normally distributed so we used nonparametric statistical tests. Categorical variables were compared with the chi-square test and continuous variables were analyzed with the Mann–Whitney U test. Overall survival (OS) was calculated as the time from diagnosis until death or the last follow-up visit. Time to thrombosis (TTT) was measured as the time from diagnosis until the first thrombotic (arterial or venous) event with patients being censored at the time of last follow-up or death, whereas time to bleeding (TTB) was measured as the time from diagnosis until the first bleeding event. Thrombotic and bleeding events present before or at the time of disease diagnosis were not taken into account for TTT and TTB calculation. Time to disease transformation (TDT) was measured as the time from disease diagnosis until the time of transformation to SMF, MDS, or AML. Survival analyses were performed with the Kaplan-Meier and the Cox regression analyses. Arterial thrombotic events considered were acute myocardial infarction, transitory ischemic attack, acute ischemic stroke, and acute peripheral arterial occlusion, whereas venous thrombotic events were defined as deep vein thrombosis and/or pulmonary embolism. Statistical calculations were performed with MedCalc Statistical Software (Medcalc Software Ltd., Ostend, Belgium, version 20.216). 

## 3. Results

### 3.1. Prevalence of Polypharmacy, PIMs, and DDIs in MPN Patients and their Clinical Correlations

A total of 124 MPN patients (ET = 63, PV = 44, MF = 9, MPN unclassifiable = 8) were included; the median age was 70 years (range 21–92) and 76 (61.3%) were females. The total number of prescriptions was 761 and the median number of prescribed medications was 5 (0–16). Hydroxycarbamide (*n* = 101, 13.2%), low-dose aspirin (*n* = 74, 9.7%), and allopurinol (*n* = 42, 5.5%) were the three most common prescriptions. A detailed list of medications prescribed to our MPN cohort is provided in Supplementary Table S1. When stratified according to the ATC system, medications with CV (C code, *n* = 269, 35%), hematopoietic (B code, *n* = 139, 18.2%), and antineoplastic and immunomodulatory effects (L code, *n* = 116, 15.2%) were found to be the most frequently used drug classes (Supplementary Table S2).

A total of 66 PIMs were identified; the most frequent PIMs were prolonged proton pump inhibitor (*n* = 15, 22.7%), tramadol (*n* = 11, 16.6%), and diazepam (*n* = 6, 9.1%) use. There was a total of 306 C interactions and the most commonly encountered were the combination of antihypertensives and loop diuretics (*n* = 43, 14%), followed by concomitant use of angiotensin-converting enzyme inhibitors (ACE-i), thiazides, and thiazide-like diuretics (*n* = 23, 7.5%), and the simultaneous use of ACE-i and salicylates (*n* = 21, 6.8%). A total of 32 D interactions were recorded; the most frequent ones were the concomitant use of warfarin and allopurinol (*n* = 5, 15.6%), combinations of opioids and central nervous system depressants (*n* = 5, 15.6%), and the simultaneous use of hydroxyurea and denosumab (*n* = 3, 9.3%). A complete list of PIMs and DDIs found in our MPN cohort with detailed explanations regarding their potential adverse health effects and pharmacodynamic DDI are provided in Table 1 and Table 2. 

Polypharmacy, at least one PIM (calculated for persons >60 years of age, *n* = 101), and at least one DDI were recorded in 76 (61.3%), 46 (45.5%) and 77 (62.1%) of patients, respectively. Seventy-four (59.6%) and 21 (16.9%) patients had at least one C or at least one D interaction, respectively. None of the patients had X interactions. Twelve patients (9.6%) were found to use ≥2 PIMs, and ≥2 C and D interactions were found in 58 (46.7%) and 6 (4.8%) patients, respectively. Eighteen patients (14.5%) used medications with both C and D interactions.

The median number of PIMs, C, and D interactions was 0 (range 0–4), 3 (range 1–20), and 1 (range 1–4), respectively. There were no statistically significant differences in the number of prescribed medications (*p* = 0.338), prevalences of polypharmacy (*p* = 0.250), PIMs (*p* = 0.857), and DDIs (*p* = 0.228) in ET vs. PV patients; we did not test for differences in MF and MPN-unclassified due to the small number of patients included. Both PIM (*p* < 0.001) and DDI (*p* < 0.001) correlated with the presence of polypharmacy.

As shown in Table 3., polypharmacy was associated with older age, prior thrombosis, presence of disease-related symptoms, oral anticoagulants, osteoarthritis/osteoporosis, autoimmune disorders, less frequent splenomegaly, and medications used for the management of CV diseases (atrial fibrillation, CHF, arterial hypertension, and hyperlipidemia). Similarly, DDI was also associated with older age, oral anticoagulants, osteoarthritis/osteoporosis, and CV disorders, whereas the use of PIMs was more frequent in patients with prior arterial thrombosis, those treated for psychiatric disorders and liver cirrhosis, and in patients using oral anticoagulants and proton pump inhibitors (*p* < 0.050 for all analyses). Finally, MPN patients with higher hemoglobin and hematocrit levels more often had D interactions; these may be caused by increased myeloproliferation and a higher disease burden and account for more frequent allopurinol, analgetic and anxiolytic use. 

### 3.2. Survival Analyses

The median follow-up time was 68 months (range 1–307). Considering the heterogeneity within MPNs and the low number of MF and MPN-unclassified patients included in the study, during survival analyses we focused solely on ET and PV patients (*n* = 108) whose OS (*p* = 0.398), TTT (*p* = 0.768), TTB (*p* = 0.629) and TDT (*p* = 0.809) did not differ. A total of 38 (35.2%) deaths (CV causes = 11, infection = 6, disease progression = 6, other/unknown = 15), 21 (19.4%) thrombotic events (14 arterial and 7 venous), 16 (14.8%) bleeding events (major gastrointestinal bleedings = 9, epistaxis = 3, prolonged bleeding after tooth extraction = 1, hemoptoa = 1, hematuria = 1, hemorrhagic shock after tonsillectomy = 1) and 10 (9.3%) disease transformations (SMF = 7, AML = 2, MDS = 1) occurred during this time. 

**Figure 2:**
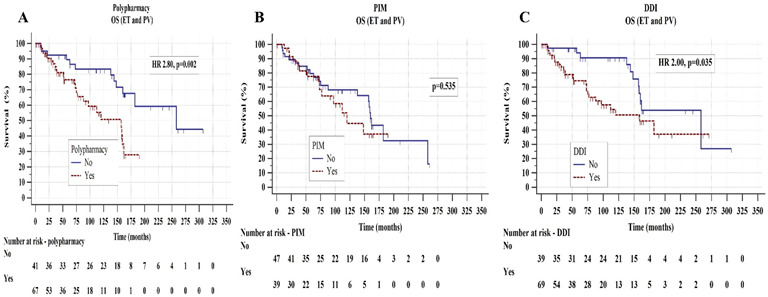
Overall survival (OS) in essential thrombocythemia (ET) and polycythemia vera (PV) patients according to polypharmacy (**A**), potentially inappropriate medications-PIM (**B**), and drug-to-drug interactions-DDI (**C**). The Kaplan-Meier and the log-rank tests were used.

Univariately, median OS was significantly shorter in an overall cohort of ET and PV patients using polypharmacy (median 157 vs. 258 months, hazard ratio-HR 2.80, *p* = 0.002) and with DDIs (median 159 vs. 258 months, HR 2.00, *p* = 0.035), whereas PIMs did not affect OS (*p* = 0.535), as shown in Figure 2**.** The associations of DDI with an inferior OS persisted for both C (HR 2.00, *p* = 0.036) and D interactions (HR 2.77, *p* = 0.067). Both polypharmacy (HR 5.22, *p* = 0.022) and DDI (HR 4.88, *p* = 0.027) remained independently associated with an inferior OS in the multivariate Cox regression models additionally adjusted for sex, high-risk disease, presence of CV risk factors, baseline leukocytosis, and cytoreductive treatment, as shown in Supplementary Table S3. 

**Figure 3:**
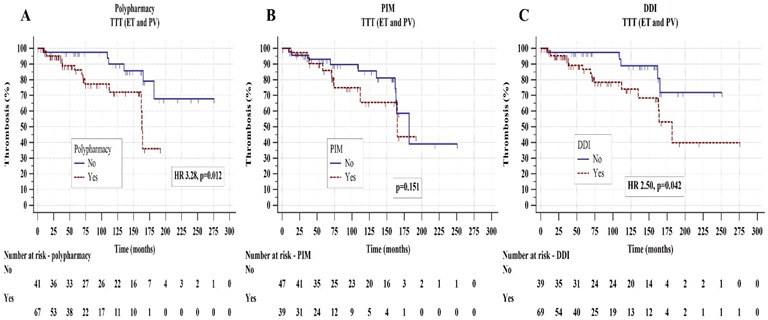
Time to thrombosis (TTT) in essential thrombocythemia (ET) and polycythemia vera (PV) patients according to polypharmacy (**A**), potentially inappropriate medications-PIM (**B**), and drug-to-drug interactions-DDI (**C**). The Kaplan-Meier and the log-rank tests were used.

Median TTT was significantly shorter in patients using polypharmacy (median 163 months vs. not reached, HR 3.28, *p* = 0.012) and with DDI (median 182 months vs. not reached, HR 2.50, *p* = 0.042) whereas there were no statistically significant differences in TTT with respect to PIM (*p* = 0.151), as shown in Figure 3**.** When analyzed separately, the association of DDD with an inferior TTT was significant for C interactions (HR 2.56, *p* = 0.036) but not for D interactions (*p* = 0.498) which could be due to a smaller number of D interactions present in the study population. In the multivariate Cox regression models adjusted for sex, high-risk disease, presence of CV risk factors, *JAK2* mutation, baseline leukocytosis, and cytoreductive treatment, polypharmacy (HR 7.60, *p* = 0.008) and DDIs (HR 5.00, *p* = 0.025) remained as predictors of an inferior TTT, as presented in Supplementary Table S4. 

There were no significant associations of polypharmacy, PIM, and DDI with respect to TTB (Supplementary Figure S1) and TDT (Supplementary Figure S2). 

Finally, we would like to point out that these survival analyses should be considered hypothesis-generating considering the absence of validation in an independent cohort. 

## 4. Discussion

To our knowledge, this is the first study to provide comprehensive details regarding the medication use in MPNs and the frequencies of polypharmacy, PIMs, and DDIs in this specific patient population. We showed that polypharmacy (61.3%), PIM (45.5%), and DDI (62.1%) are very frequent among MPN patients. In fact, polypharmacy seems to be significantly higher than in the general population (39% in persons ≥65 years of age) [26] and, together with DDI, is mostly associated with the management of disease-related symptoms in MPNs, osteoarthritis/osteoporosis, and different CV disorders, i.e., atrial fibrillation, CHF, arterial hypertension, hyperuricemia, or hyperlipidemia. These observations again highlight the significant symptom burden and the importance of appropriate CV risk management in MPN patients. 

The most common PIM was prolonged proton pump inhibitor use—this may be related to the fact that MPN patients have been shown to frequently suffer from dyspepsia, *Helicobacter pylori* infection, and peptic ulcer disease [38,39]. Considering that PIM also correlated with the presence of prior thrombosis and anticoagulant use, this could suggest that many MPN patients receive prolonged proton pump inhibitor treatment together with aspirin and/or oral anticoagulants due to already present dyspeptic symptoms and/or because of physicians’ fear of future adverse gastrointestinal events. These observations may be even more important in the light of recent evidence suggesting suboptimal platelet inhibition in ET with once-daily low-dose aspirin when compared to more intensive aspirin regimens which may also cause more abdominal discomfort [40]. On the other hand, even though prolonged pump inhibitors were the most common PIM, none of the MPN patients experienced *Clostridium difficile* infection. Other common PIMs were tramadol and diazepam. It should be pointed out that many MPN patients suffer from anxiety and depression [41], warranting the use of anxiolytics. On the other hand, osteoarthritis [42] and osteoporosis [43] have been shown to be a frequent feature in MPNs, and tramadol and/or nonsteroidal anti-inflammatory drugs used for their treatment could be involved in a significant proportion of PIMs and DDIs. Specifically, tramadol and diazepam may cause synergistic depressive effects on the central nervous system and be responsible for adverse health outcomes. In addition, we found that diazepam and other anxiolytics were often chronically used by MPN patients which is not standard practice due to their addictive effect. This suggests that many MPN patients may indeed suffer from different cumbersome disease-related symptoms (i.e., itching, anxiety, or insomnia) warranting such treatment for symptom relief. Finally, the use of PIMs was not associated with inferior outcomes in MPNs, possibly due to the fact that proton pump inhibitors were the most common PIMs, and these compounds are usually considered to be relatively safe medications.

Even though there are no guidelines to suggest its use in MPN patients, allopurinol was frequently prescribed to MPN patients and its interactions with warfarin and loop diuretics were often found. Allopurinol is a drug often used in MPN patients due to baseline hyperuricemia or because of fear of anticipated hyperuricemia caused by an increased cell turnover during cytoreductive treatment. More importantly, hyperuricemia was also recently shown to be associated with inferior outcomes MPNs [16,17]. Therefore, considering that a significant proportion of MPN patients may suffer from thrombotic events, arterial hypertension, or CHF, necessitating the use of warfarin or diuretics, future studies are warranted to fully elucidate the role of serum uric acid in the pathogenesis of thrombosis in MPNs and whether the use of allopurinol may have a beneficial effect on different disease-related outcomes. This may be even more important when considering the allopurinol-related DDIs and the fact that the vast majority of MPN patients do not have gout and have well-controlled serum uric acid levels, thus questioning the role of continuous allopurinol use. Other common DDIs were the combinations of ACE-i and diuretics, or the simultaneous use of ACE- and salicylates, potentially having synergistic hypotensive and nephrotoxic effects, respectively. In addition to their negative effects on the CV system due to synergistic hypotensive properties, these DDI may also cause worsening of kidney function which has been associated with inferior outcomes in MPNs [18,19,20]. On the other hand, the use of ACE-i has been shown to have renoprotective properties in PV [44], suggesting that the hypotensive CV effects could be the more detrimental ones. 

The adverse health effects of polypharmacy and DDI were also confirmed in survival analyses. Both polypharmacy and DDI were shown to be predictors of an inferior OS, independently of high-risk disease and the presence of CV risk factors. This important observation suggests that inadvertent DDI due to multiple medication use may be responsible for the inferior outcomes in a subset of MPN patients. Moreover, both polypharmacy and DDI predicted an inferior TTT rendering other clinically relevant risk factors insignificant during multivariate analyses, most probably due to their overlapping prognostic properties. Nevertheless, these results may provide an important danger signal regarding the potential risks of combining drugs with pharmacodynamic interactions in MPN patients. 

Even though combinations of warfarin-allopurinol and aspirin-salicylates DDI were frequently encountered in our MPN cohort and the fact that their pharmacodynamic synergism may potentially cause an increased risk of bleeding, we did not observe such associations. This may be because MPN patients are more prone to thrombosis than the general population, countering the potentially negative effect of these interactions. In addition, the presented cohort had a very low number of *CALR*-mutated patients whose thrombotic risk is much lower than that of their *JAK2*-mutated counterparts [1]. No effect of polypharmacy, PIM use, and DDI was seen regarding the disease transformation risk. It is noteworthy that hydroxyurea, a cytoreductive medication most often used to treat MPNs in this patient cohort, does not have a large number of clinically significant DDI [37]. Even though disease-modifying properties and thus attractiveness of the use of hydroxyurea and other cytoreductive drugs are still debated, the absence of significant DDI may provide further reassurance to physicians regarding its safety and efficacy in the treatment of MPNs.

The limitations of this study are its retrospective single-center design and the limited number of patients included. Additionally, due to the small number of MF patients, we could only assess the prognostic impact of polypharmacy, PIM, and DDI in ET and PV patients. Therefore, future studies should focus also on MF patients. Nevertheless, this study provided important signals regarding the potential risks of polypharmacy and DDI in MPN patients and it may alert the clinicians caring for MPNs to stay vigilant and tactful in managing disease-related symptoms and to periodically reassess medications used to treat patients’ other clinical conditions, especially CV disorders. Shared decision-making by physicians and MPN patients should be implemented in order to avoid medication overuse and potentially inadvertent DDI.

## Figures and Tables

**Table 1:** The list of potentially inappropriate medications (PIM) in patients with chronic myeloproliferative neoplasms.

PIMs	Total Number of PIMs = 66	Main Reason
Proton pump inhibitors (>8 weeks): pantoprazole, esomeprazole	15 (22.7%)	Long-term high-dose proton pump inhibitor therapy is associated with an increased risk of *Clostridium difficile* infection and hip fracture. Inappropriate if used >8 weeks in maximal dose without clear indication.
Tramadol	11 (16.6%)	More adverse effects in older adults; central nervous system side effects such as confusion, vertigo, and nausea.
Diazepam	6 (9.1%)	Risk of falling with hip fracture; prolonged reaction times; psychiatric reactions (can also be paradoxical, e.g., agitation, irritability, hallucinations, psychosis); cognitive impairment; depression.
Rivaroxaban	5 (7.6%)	Limited information on use for older adults; risk of bleeding events; potential unavailability of a reversal agent in case of overdose; risk of bleeding may be higher in cases of severe renal failure.
Alprazolam	3 (4.5%)	Risk of falling with hip fracture; prolonged reaction times; psychiatric reactions (can also be paradoxical, e.g., agitation, irritability, hallucinations, psychosis); cognitive impairment; depression.
Ranitidine	3 (4.5%)	Central nervous adverse effects, including confusion.
Bromazepam	2 (3%)	Risk of falling with hip fracture; prolonged reaction times; psychiatric reactions (can also be paradoxical, e.g., agitation, irritability, hallucinations, psychosis); cognitive impairment; depression.
Theophylline	2 (3%)	Higher risk of central nervous system stimulant effects.
Maprotiline	2 (3%)	Peripheral anticholinergic side effects (e.g., constipation, dry mouth, orthostatic hypotension, cardiac arrhythmia); central anticholinergic side effects (drowsiness, inner unrest, confusion, other types of delirium); cognitive deficit; increased risk of falling.
Iron supplements > 325 mg	2 (3%)	Doses > 325 mg/day do not considerably increase the amount absorbed but greatly increase the incidence of constipation.
Etoricoxib	1 (1.5%)	Very high risk of gastrointestinal bleeding, ulceration, or perforation, which may be fatal; cardiovascular contraindications.
Indomethacin	1 (1.5%)	Very high risk of gastrointestinal bleeding, ulceration, or perforation, which may be fatal;risk of central nervous system disturbances.
Ketoprofen	1 (1.5%)	Very high risk of gastrointestinal bleeding, ulceration, or perforation, which may be fatal.
Pramipexole	1 (1.5%)	Side effects include orthostatic hypotension, gastrointestinal tract symptoms, hallucinations, confusion, insomnia, peripheral edema.
Metildigoxine	1 (1.5%)	Elevated glycoside sensitivity (women > men); risk of intoxication.
Verapamil	1 (1.5%)	May worsen constipation; risk of bradycardia.
Moxonidine	1 (1.5%)	Risk of orthostatic hypotension, bradycardia, syncope, and central nervous system side effects (sedation, depression, cognitive impairment).
Amiodarone	1 (1.5%)	Associated with QT interval problems and risk of provoking torsades de pointes.Data suggest that for most older adults rate control yields a better balance of benefits and harms than rhythm control.
Propafenone	1 (1.5%)	High risk of drug interactions.Data suggest that for most older adults rate control yields a better balance of benefits and harms than rhythm control.
Pentoxifylline	1 (1.5%)	No proven efficacy; unfavorable risk/benefit profile; orthostatic hypotension and fall risks are increased with most vasodilators.
Carbamazepine	1 (1.5%)	Increased risk of SIADH-like syndrome; adverse events like carbamazepine-induced confusion and agitation, atrioventricular block, and bradycardia.
Sitagliptin	1 (1.5%)	Limited safety data are available for adults aged ≥ 75 years old. Subjects aged 65 to 80 years had higher plasma concentrations than younger subjects. Risk of hypoglycemia, dizziness, headache, and peripheral edema.
Solifenacin	1 (1.5%)	Anticholinergic side effects (e.g., constipation, dry mouth, central nervous system side effects); electrocardiogram changes (prolonged QT).
Aluminum hydroxide	1 (1.5%)	Renal excretion of aluminum decreases in older individuals. Risk of central nervous system toxicity.
Magnesium hydroxide	1 (1.5%)	Risk of hypermagnesemia, which is higher in moderate to severe renal failure.

**Table 2:** The list of drug-to-drug interactions (DDI) in patients with chronic myeloproliferative neoplasms.

D Category (Modify Regimen)	Interaction	Total Number of D Category DDI = 32
warfarin—allopurinol	Allopurinol may enhance the anticoagulant effect of vitamin K antagonists.	5 (15.6%)
OPIOID AGONISTS—CENTRAL NERVOUS SYSTEM DEPRESSANTS tramadole- bromazepam/ oxazepam/diazepam/ alprazolam/maprotiline	Central nervous system depressants may enhance the central nervous system depressant effect of opioid agonists.	5 (15.6%)
hydroxyurea—denosumab	Denosumab may enhance the immunosuppressive effects of immunosuppressants.	3 (9.3%)
NONSTEROIDAL ANTI- INFLAMMATORY DRUGS— LOOP DIURETICS etorocoxib—furosemide indomethacin—torasemide	Nonsteroidal anti-inflammatory agents may diminish the diuretic effect of loop diuretics. Loop diuretics may enhance the nephrotoxic effect of nonsteroidal anti-inflammatory agents.	2 (6.25%)
SALICYLATES—NONSELECTIVE NONSTEROIDAL ANTI- INFLAMMATORY DRUGS aspirin—ibuprofen aspirin -ketoprofen	Nonsteroidal anti-inflammatory agents (nonselective) may enhance the adverse/toxic effect of salicylates. An increased risk of bleeding may be associated with the use of this combination. Nonsteroidal anti-inflammatory agents (nonselective) may diminish the cardioprotective effect of salicylates. Salicylates may decrease the serum concentration of nonsteroidal anti-inflammatory agents.	2 (6.25%)
simvastatin—amlodipine	Amlodipine may increase the serum concentration of simvastatin, which is associated with a significant increase in the risk for adverse muscle effects; the dose of simvastatin must be limited to 20 mg if coadministering with amlodipine.	2 (6.25%)
BETA BLOCKERS— ALPHA2-AGONISTS (bisoprolol—brimonidine, timolol—brimonidine)	Alpha2-agonists may enhance the AV-blocking effect of beta-blockers. Sinus node dysfunction may also be enhanced. Beta-blockers may enhance the rebound hypertensive effect of alpha2-agonists when they are abruptly withdrawn. Ophthalmic beta-blockers likely pose a reduced risk.	2 (6.25%)
insulin—dapagliflozin	SGLT2 inhibitors may enhance the hypoglycemic effect of insulins.	2 (6.25%)
insulin—linagliptin	Dipeptidyl peptidase-4 inhibitors may enhance the hypoglycemic effect of insulins.	1 (3.1%)
insulin—dulaglutide	Glucagon-like peptide-1 agonists may enhance the hypoglycemic effect of insulins.	1 (3.1%)
warfarin—indomethacin	Nonselective anti-inflammatory agents may enhance the anticoagulant effect of vitamin K antagonists because they affect platelet aggregation, increasing the risk of gastrointestinal bleeding.	1 (3.1%)
warfarin—amiodarone	Amiodarone may enhance the anticoagulant effect of vitamin K antagonists. Amiodarone may increase the serum concentration of vitamin K antagonists.	1 (3.1%)
simvastatine—amiodarone	Amiodarone may increase serum concentrations of the active metabolite(s) of simvastatin. Amiodarone may increase the serum concentration of simvastatin.	1 (3.1%)
verapamil—atorvastatin	Atorvastatin may increase the serum concentration of verapamil. Verapamil may increase the serum concentration of atorvastatin.	1 (3.1%)
prednisone—potassium	Antacids may decrease the bioavailability of oral corticosteroids.	1 (3.1%)
iron sulphate—potassium	Antacids may decrease the absorption of iron preparations.	1 (3.1%)
risedronate—calcium carbonate	Polyvalent cation-containing products may decrease the serum concentration of bisphosphonate derivates.	1 (3.1%)
**C category** (**monitor therapy**)	**Interaction**	**Total number of C category DDI,** ***n* = 306**
ANTIHYPERTENSIVE AGENTS -LOOP DIURETICS (e.g., bisoprolol—furosemide, hydrochlorothiazide—furosemide, amlodipine—furosemide, lercanidipin—furosemide, and similar combinations)	Loop diuretics may enhance the hypotensive effect of antihypertensive agents.	43 (14%)
ACE-I—THIAZIDE AND THIAZIDE-LIKE DIURETICS (e.g., lisinopril—hydrochlorothiazide, perindopril—indapamide, ramipril—hydrochlorothiazide, and similar combinations)	Thiazide and thiazide-like diuretics may enhance the hypotensive and nephrotoxic effect of ACE inhibitors.	23 (7.5%)
ACE-I—SALICYLATES (perindopril—aspirin, ramipril—aspirin, lisinopril—aspirin)	Salicylates may enhance the neprotoxic effect of ACE inhibitors. Salicylates may diminish the therapeutic effect of ACE inhibitors.	21 (6.8%)
ANTIDIABETIC AGENTS— BETA-BLOCKERS (metformin—bisoprolol, insulin bisoprolol, dapagliflozin—bisoprolol, and similar combinations)	Beta-blockers may enhance the hypoglycemic effect of antidiabetic agents.	16 (6.8%)
ALLOPURINOL—LOOP DIURETICS (allopurinol—furosemide/torasemide)	Loop diuretics may enhance the adverse/toxic effects of allopurinol. Loop diuretics may increase the serum concentration of allopurinol, specifically the concentration of its active metabolite oxypurinol.	15 (4.9%)
SALICYLATES—LOOP DIURETICS (aspirin—furosemide, aspirin—torasemide)	Salicylates may diminish the therapeutic effect of loop diuretics. Loop diuretics may increase the serum concentration of salicylates.	14 (4.5%)
ANTIDIABETIC AGENTS—HYPERGLYCEMIA-ASSOCIATED AGENTS (insulin—furosemide, metformine—hydrochlorothiazide, metformin—furosemide, and similar combinations)	Hyperglycemia-associated agents may diminish the the therapeutic effect of antidiabetic agents.	14 (4.5%)
ALLOPURINOL—ACE INHIBITORS (Allopurinol—lisinopril/ramipril/ perindopril)	ACE inhibitors may enhance the potential for allergic or hypersensitivity reactions to allopurinol.	13 (4.2%)
ALLOPURINOL—THIAZIDE AND THIAZIDE-LIKE DIURETICS (allopurinol -hydrochlorothiazide/ indapamide)	Thiazide and thiazide-like diuretics may enhance the potential for allergic or hypersensitivity reactions to allopurinol.	12 (3.9%)
HYPOTENSION-ASSOCIATED AGENTS—BLOOD PRESSURE LOWERING AGENTS (amiodarone—furosemide, amiodarone—hydrochlorothiazide, amiodarone—lisinopril, bisoprolol -levodopa)	Blood pressure-lowering agents may enhance the hypotensive effect of hypotension-associated agents.	12 (3.9%)
warfarin—tramadol warfarin—paracetamol warfarin—rosuvastatin warfarin—simvastatin warfarin—prednisone warfarin—levothyroxine	Drugs that may enhance the anticoagulant effect of warfarin.	11 (3.6%)
ACE INHIBITORS—LOOP DIURETICS (furosemide—lisinopril, furosemide—perindopril)	Loop diuretics may enhance the hypotensive and nephrotoxic effect of ACE inhibitors.	8 (2.6%)
DIURETICS—OPIOID AGONISTS (furosemide—tramadole, Hydrochlorothiazide— tramadole, indapamide—tramadol)	Opioid agonists may enhance the adverse/toxic effects of diuretics. Opioid agonists may diminish the therapeutic effect of diuretics.	8 (2.6%)
METFORMIN—ACE INHIBITORS (metformin-ramipril/perindopril/ lisinopril)	ACE inhibitors may enhance the adverse/toxic effects of metformin. This includes both a risk for hypoglycemia and lactic acidosis.	4 (1.3%)
warfarin—torasemide warfarin—esomeprazole warfarin—propafenone	Drugs that may increase the serum concentration of warfarin.	3 (0.9%)

**Table 3:** The associations of polypharmacy, potentially inappropriate medications (PIM) drug-to-drug interactions (DDI) with different clinical and laboratory characteristics in patients with chronic myeloproliferative neoplasms. The chi-square and the Mann–Whitney U tests were used.

Variable	Overall (*n* = 124)	Polypharmacy (*n* = 76, 61.3%)	At Least One PIM (*n* = 46, 38.7%)	At Least One DDI-overall (*n* = 77, 62.1%)	At Least One DDI-C (*n* = 74, 61.2%)	At Least One DDI-D (*n* = 21, 16.9%)
Age, years (median, range)	70 (21–92)	72 * vs. 64, ***p* = 0.001**	76 * vs. 71, ***p* < 0.001**	72 * vs. 66, ***p* = 0.010**	72 * vs. 66, ***p* = 0.013**	78 * vs. 66, ***p* < 0.001**
Sex, female	76 (61.3%)	74% * vs. 40.4%, ***p* < 0.001**	*p* = 0.208	*p* = 0.068	*p* = 0.070	*p* = 0.125
ET PV MF MPN-u	63 (50.8%) 44 (35.5%) 9 (7.3%) 8 (6.5%)	*p* = 0.689	*p* = 0.130	*p* = 0.532	*p* = 0.573	*p* = 0.519
*JAK2* mutated *CALR* mutated Negative/Unknown	86 (69.4%) 7 (5.6%) 31 (25%)	*p* = 0.581	*p* = 0.159	*p* = 0.327	*p* = 0.355	*p* = 0.909
Palpable splenomegaly (*n* = 122)	36 (29.5%)	21.6% * vs. 41.7% *, ***p* = 0.018**	*p* = 0.532	*p* = 0.386	*p* = 0.372	*p* = 0.958
Prior thrombosis -arterial -venous	28 (22.6%) 16 (57.1%) 12 (42.8%)	28.9% *, vs. 12.5%, ***p* = 0.033** *p* = 0.601	34.8% *, vs. 16.4%, ***p* = 0.033** *p* = 0.617	*p* = 0.249 *p* = 0.722	*p* = 0.267 *p* = 0.827	*p* = 0.197 *p* = 0.386
Disease-related symptoms	35 (28.2%)	35.5% * vs. 16.7%, ***p* = 0.023**	*p* = 0.883	*p* = 0.353	*p* = 0.287	*p* = 0.103
Arterial hypertension	85 (68.5%)	85.5% * vs. 41.7%, ***p* < 0.001**	*p* = 0.481	84.4% * vs. 42.%. *p* < 0.001	85.1% * vs. 41.7%, ***p* < 0.001**	*p* = 0.064
Diabetes mellitus (*n* = 122)	15 (12.3%)	*p* = 0.315	*p* = 0.271	*p* = 0.348	*p* = 0.309	*p* = 0.253
Hyperlipidemia	45 (36.3%)	46.1% * vs. 20.8%, ***p* = 0.004**	*p* = 0.613	45.5% * vs. 21.3%, ***p* = 0.006**	**44.6% vs. 20.8%,** ***p* = 0.009**	*p* = 0.494
Chronic kidney disease (*n* = 101)	15 (14.9%)	*p* = 0.227	*p* = 0.255	*p* = 0.198	*p* = 0.179	*p* = 0.624
Chronic heart failure	32 (26.4%)	39.2% * vs. 6.4%, *p* < 0.001	*p* = 0.638	38.7% * vs. 6.5%, *p* < 0.001	39.7% * vs. 6.5%, *p* = 0.001	50% * vs. 21.8%, ***p* = 0.009**
Atrial fibrillation	18 (14.5%)	19.7% * vs. 6.2%, *p* = 0.038	*p* = 0.230	19.5% * vs. 6.4%, *p* = 0.045	20.3% * vs. 6.2%, *p* = 0.033	28.6% * vs. 11.7%, ***p* = 0.045**
Peptic ulcer disease	16 (12.9%)	*p* = 0.322	22.9% * vs. 6.6%, *p* = 0.008	*p* = 0.106	*p* = 0.126	*p* = 0.836
Autoimmune disorders -thyroiditis = 5 -ulcerative colitis = 4 -rheumatoid arthritis = 4 -Raynaud syndrome with undetermined collagenosis = 1	12 (9.7%)	14.5% * vs. 2.1%, *p* = 0.023	*p* = 0.529	*p* = 0.732	*p* = 0.681	*p* = 0.435
Osteoarthritis/ osteoporosis	18 (14.5%)	19.7% * vs. 6.2%, *p* = 0.038	*p* = 0.351	19.5% * vs. 6.4%, *p* = 0.045	*p* = 0.108	33.3% * vs. 10.7%, ***p* = 0.007**
Pulmonary diseases -COPD = 7 -asthma 2 -interstitial pulmonary disease = 1	10 (8%)	*p* = 0.867	*p* = 0.529	*p* = 0.590	*p* = 0.638	*p* = 0.469
Neurological disorders -Parkinson’s disease = 2 -dementia = 2 -migraine = 1 -vertigo = 1 -epilepsy = 1	7 (6.5%)	*p* = 0.572	8.7% * vs. 0%, ***p* = 0.026**	9.1% * vs. 0%, ***p* = 0.034**	9.5% * vs. 0%, ***p* = 0.030**	*p* = 0.848
Psychiatric disorders -alcoholism = 3 -depression = 2 -psychoorganic syndrome = 2 -autism = 1 -anxiety = 1	9 (6.5%)	*p* = 0.732	13% * vs. 1.8%, *p* = 0.027	*p* = 0.315	*p* = 0.289	19% * vs. 4.9%, *p* = 0.022
Hyperuricemia	11 (8.9%)	*p* = 0.144	*p* = 0.056	13% * vs. 2.1%, *p* = 0.039	12.2% * vs. 2.1%, *p* = 0.048	*p* = 0.340
Liver cirrhosis	7 (5.6%)	*p* = 0.173	13% * vs. 1.8%, *p* = 0.027	*p* = 0.186	*p* = 0.254	*p* = 0.848
Smoking (active/prior vs. never)	9 (7.3%)	*p* = 0.293	*p* = 0.229	*p* = 0.770	*p* = 0.725	*p* = 0.629
Other malignancy -colon cancer = 1 -monoclonal gammopathy of undetermined significance = 1	2 (1.6%)	-	-	-	-	-
Cytoreduction -hydroxyurea -ruxolitinib -anagrelide -interferons	102 82.3%) 88 (86.2%) 6 (5.8%) 5 (4.9%) 3 (2.9%)	*p* = 0.094	*p* = 0.345	*p* = 0.870	*p* = 0.938	*p* = 0.864
Aspirin	75 (60.5%)	*p* = 0.990	*p* = 0.078	*p* = 0.177	*p* = 0.166	*p* = 0.406
Oral anticoagulants	22 (18%)	27% * vs. 4.2%, *p* = 0.001	31.1% * vs. 13%, *p* = 0.007	25% * vs. 6.5%, *p* = 0.010	24.7% * vs. 6.4%, *p* = 0.011	**42.9% vs. 12.9%,** ***p* = 0.001**
Total leukocytes (×10^9^/L)	9.2 (4.4–156.6)	*p* = 0.280	*p* = 0.726	*p* = 0.312	*p* = 0.303	*p* = 0.105
Hemoglobin (g/L)	143 (48–229)	*p* = 0.529	*p* = 0.397	*p* = 0.761	*p* = 0.923	**151 vs. 141,** ***p* = 0.010**
Hematocrit	46.2 (14.4–90)	*p* = 0.340	*p* = 0.415	*p* = 0.633	*p* = 0.734	**48.9 vs. 45.9,** ***p* = 0.005**
Platelets (×10^9^/L)	547 (40–3211)	*p* = 0.204	*p* = 0.419	*p* = 0.180	*p* = 0.204	*p* = 0.201
LDH (IU/L)	246 (136–529)	*p* = 0.301	*p* = 0.823	*p* = 0.358	*p* = 0.307	*p* = 0.787

Statistically significant *p* values are bolded and set at <0.050. * indicating variables of interest. PIMs = potentially inappropriate medications, DDsI = drug-to-drug interactions, ET = essential thrombocythemia, PV = polycythemia vera, MF = myelofibrosis, MPN-u = myeloproliferative neoplasm-unclassified, *JAK2* = Janus Kinase 2, *CALR* = calreticulin, LDH = lactate dehydrogenase.

## Data Availability

Data are available from the corresponding author upon reasonable request.

## Supplementary Materials

The following supporting information can be downloaded at: https://www.mdpi.com/article/10.3390/biomedicines11051301/s1, Table S1: Total number of drug precriptions; Table S2; Total number of prescribed medications according to Anatomical Therapeutic Chemical Classification (ATC) system; Table S3: Multivariate Cox regression analysis of factors associated with overall survival. DDI = drug to drug interactions, CV = cardiovascular, PV = polycythemia vera, HR = hazard ratio; Table S4: Multivariate Cox regression analysis of factors associated with time to thrombosis. DDI = drug to drug interactions, CV = cardiovascular, *JAK2* = Janus Kinase 2, HR = hazard ratio; Figure S1: Time to bleeding (TTB) in essential thrombocythemia (ET) and polycythemia vera (PV) patients according to polypharmacy (A), potentially inappropriate medications-PIM (B) and drug-to-drug interactions (C). The Kaplan-Meier and the log-rank tests were used; Figure S2: Time to disease transformation (TDT) in essential thrombocythemia (ET) and polycythemia vera (PV) patients according to polypharmacy (A), potentially inappropriate medications-PIM (B) and drug-to-drug interactions (C). The Kaplan-Meier and the log-rank tests were used.
