# Supplementary material for: Polypharmacy, Potentially Inappropriate Medications, and Drug-to-Drug Interactions in Patients with Chronic Myeloproliferative Neoplasms
Source: Biomedicines. 2023 Apr 27;11(5):1301. doi: 10.3390/biomedicines11051301 (PMC10215953; doi:10.3390/biomedicines11051301)
Supplement: Supplementary file 1 [file biomedicines-11-01301-s001.zip › biomedicines-2330090-supplementary.pdf]

Table S1. Total number of drug prescriptions.

Compound	n=761
Hydroxycarbamide	101
Low-dose aspirin	74
Allopurinol	42
Bisoprolol	39
Folic acid	33
Furosemide	30
Hydrochlorothiazide	23
Pantoprazole	21
Atorvastatin	19
Amlodipine	20
Warfarin	18
Ramipril	17
Paracetamol	16
Lisinopril	13
Tramadole	13
Perindopril	10
Indapamide	9
Simvastatine	9
Insulin	9
Lercanidipine	9
Levothyroxine	9
Metformin	8
Rosuvastatin	8

Esomeprazole	7
Losartan	7
Rivaroxaban	7
Ruxolitinib	6
Nebivolol	6
Cholecalciferole	6
Diazepam	6
Anagrelide	5
Potassum chloride/citrate	5
Carvedilol	5
Valasartan	5
Alprazolam	5
Iron supplements	5
Isosorbide mononitrate	4
Atenolol	4
Lacidipine	4
Trandolapril	4
Prednisone	4
Ranitidine	4
Mesalazine	4
Tamsulozin	4
Interferons	3
Oksazepam	3
Torasemide	3
Denosumab	3
Fluticasone	3
Calcium polystyrene sulphonate	3

Methylprednisolone	2
Clopidogrel	2
Ibuprofen	2
Dapagliflozin	2
Linagliptin	2
Calcium carbonate	2
Amiodarone	2
Moxonidine	2
Cilazapril	2
Fenofibrate	2
Carbamazepine	2
Pramipexole	2
Maprotiline	2
Bromazepam	2
Salbutamole	2
Salmeterole	2
Teophylline	2
Timolol	2
Ciclesonide	2
Acyclovir	2
Propafenone	2
Etoricoxib	1
Amyloride	1
Ketoprofen	1
Indomethacine	1
Bikalutamide	1
Sodium risedronate	1

Calcitriol	1
Sitagliptine	1
Glicvidone	1
Repaglinide	1
Gliclazide	1
Nitroglycerine	1
Metildigoxin	1
Urapidil	1
Dulaglutide	1
Baking soda	1
Aluminum hydroxide - magnesium carbonate/magnesium hydroxyide	1
Pancreatic enzymes	1
Pentoxifylline	1
Felodipine	1
Verapamil	1
Diltiazem	1
Telmisartan	1
Methadone	1
Pregabaline	1
Phenobarbital	1
Clonazepam	1
Carbidopa/Levodopa	1
Benserazide	1
Haloperidole	1
Tianeptine	1
Betahistine	1

Cinnarizine	1
Solifenacin	1
Finasteride	1
Brimonidine	1
Dorzolamide	1
Brinzolamide	1
Latanoprost	1
Spironolactone	1
Sevelamer	1
Propranolol	1
Ursodeoxycholic acid	1
Izoniazide	1
Sulfamethoxazole Trimethoprim	1

Table S2. Total number of prescribed medications according to Anatomical Therapeutic Chemical Classification (ATC) system.

**Code**	**n=761**
**A** (Alimentary tract and metabolism)	80 (10.5%)
**B (**Blood and blood forming organs)	139 (18.2%)
**C** (Cardiovascular system)	269 (35%)
**D** (Dermatologicals)	0
**G** (Genitourinary system and sex hormones)	6 (0.7%)
**H** (Systemic hormonal preparations, excluding sex hormones and insulins)	15 (1.9%)
**J** (Antiinfectives for systemic use)	3 (3.9%)
**L** (Antineoplastic and immunomodulating agents)	116 (15.2%)
**M** (Musculo-skeletal system)	51 (6.7%)
**N** (Nervous system)	61 (8%)
**P** (Antiparasitic products, insecticides and repellents)	0
**R** (Respiratory system)	11 (1.4%)
**S** (Sensory organs)	6 (0.7%)
**V** (various)	4 (0.5%)

Figure S1. Time to bleeding (TTB) in essential thrombocythemia (ET) and polycythemia vera (PV) patients according to polypharmacy (A), potentially inappropriate medications-PIM (B) and drug-to-drug interactions-DDI (C). The Kaplan-Meier and the log-rank tests were used.

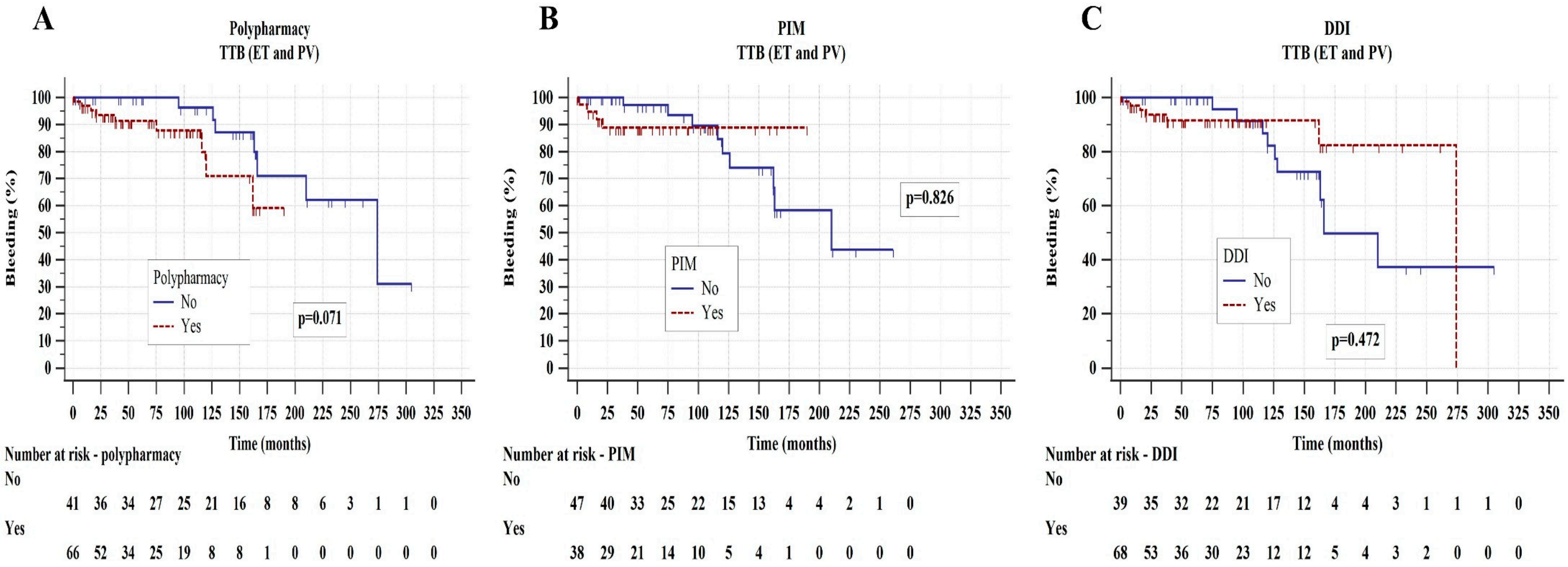

Figure S2. Time to disease transformation (TDT) in essential thrombocythemia (ET) and polycythemia vera (PV) patients according to polypharmacy (A), potentially inappropriate medications-PIM (B) and drug-to-drug interactions-DDI (C). The Kaplan-Meier and the log-rank tests were used.

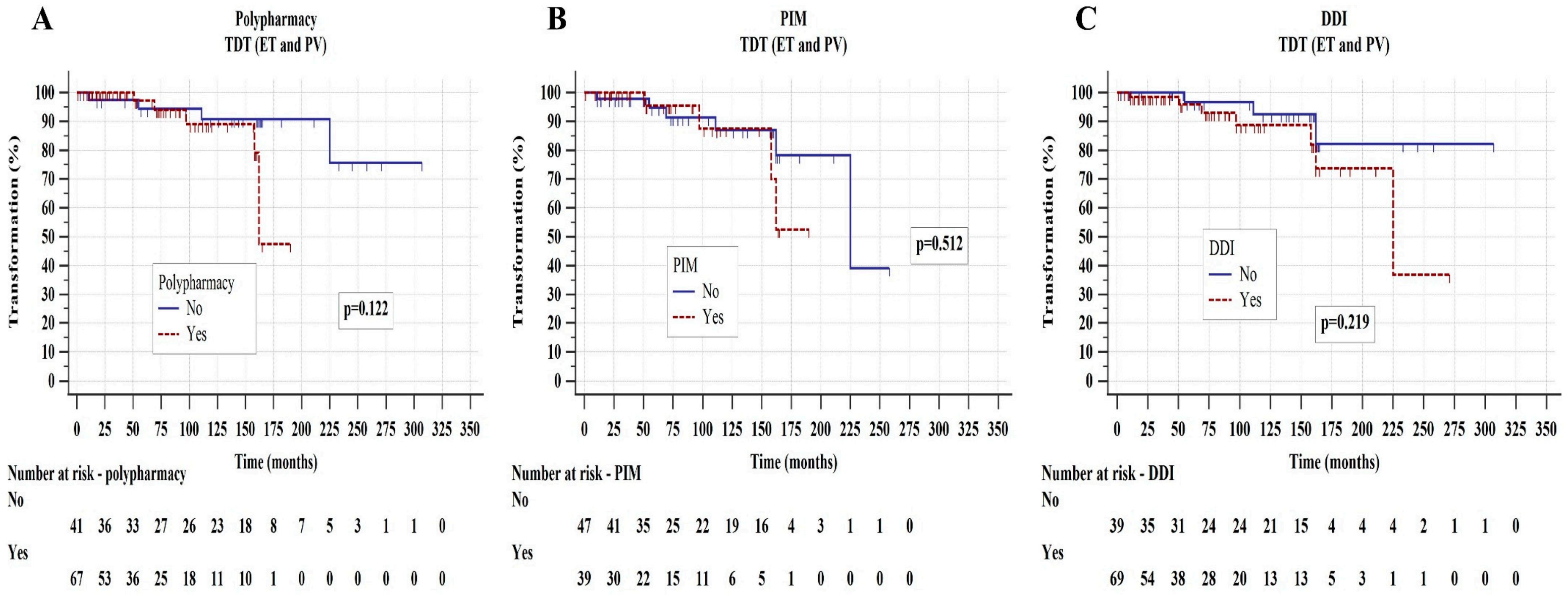

Table S3. Multivariate Cox regression analysis of factors associated with overall survival. DDI=drug to drug interactions, CV=cardiovascular, PV=polycythemia vera, HR=hazard ratio.

	**Polypharmacy**	**DDI**	**Sex**	**High-risk disease**	**CV risk factors**	**PV phenotype**	**Leukocytes>11x10^9/L**	**Cytoreduction**
Model 1	**HR 5.22, p=0.022**	-	HR 0.08, p=0.767	**HR 5.09 p=0.023**	**HR 8.71, p=0.003**	HR 1.24, p=0.263	HR 0.05, p=0.807	HR 0.07, p=0.787
Model 2	-	**HR 4.88, p=0.027**	HR 0.03, p=0.856	**HR 5.01, p=0.025**	**HR 8.58, p=0.003**	HR 3.73, p=0.053	HR 0.01, p=0.971	HR 0.56, p=0.450

Statistically significant *p* values are bolded and set at < 0.050.

Table S4. Multivariate Cox regression analysis of factors associated with time to thrombosis. DDI=drug to drug interactions, CV=cardiovascular, JAK2=Janus Kinase 2, HR=hazard ratio.

	**Polypharmacy**	**DDI**	**Sex**	**High-risk disease**	**CV risk factors**	**JAK2 mutation**	**Leukocytes >11x10^9/L**	**Cytoreduction**
Model 1	**HR 7.60, p=0.008**	-	HR 3.30, p=0.069	HR 0.50, p=0.476	HR 2.64, p=0.103	HR 0.16, p=0.688	HR 1.55, p=0.212	HR 0.24, p=0.617
Model 2	-	**HR 5.00, p=0.025**	HR 1.20, p=0.273	HR 0.18, p=0.670	HR 2.12, p=0.145	HR 0.10, p=0.751	HR 1.34, p=0.246	HR 1.27, p=0.259

Statistically significant *p* values are bolded and set at < 0.050.
